# The Work of the University Centre of Cardiac Research, 1927-31

**Published:** 1931

**Authors:** Carey F. Coombs

**Affiliations:** Physician, Bristol General Hospital


### THE WORK OF THE UNIVERSITY CENTRE OF CARDIAC RESEARCH, 1927–31.

BY

Carey F. Coombs, M.D., F.R.C.P.,

*Physician, Bristol General Hospital.*

---

In 1926 a Sub-committee of the Science Committee of the British Medical Association, appointed "to inquire into the steps which have been, and are being, taken in relation to the prevention, detection, and treatment of cardiac disease in children, and to make proposals in connexion therewith," and presided over by Sir Humphry Rolleston, Bart., decided to institute inquiries into the environment in which juvenile rheumatism was most prone to develop. One of these inquiries, it was thought, should concern itself with an area that included a wide variety of social and geographical conditions, and it was decided that these requirements would be met by taking Bristol as a centre and the three counties of Gloucestershire, Somerset and Wilts as the periphery. The writer, who was a member of that Sub-committee, accepted the responsibility of organizing this inquiry, the initial stages of which were made much easier by the kind offices of Sir Humphry Rolleston and the Secretary of the Sub-committee, Dr. Reginald Miller. Thanks to these, a grant in aid of clerical expenses was secured from the Medical Research Council, and valuable advice and help were also given by Sir George Newman.

Next, it became necessary to secure premises to serve as an office and incidentally as a laboratory and a clinic. These were generously provided by the Bristol General Hospital Committee in the octagon room on the first floor, a room used for nurses' cubicles until the opening of the Susan Britton Wills Nurses' Home set it free, but remembered by an older generation as the Hospital Chapel. This was equipped by a loan from the University of Bristol, and at their wish entitled "the University Centre of Cardiac Research." The necessary technical assistance was secured through a generous grant from the R. L. St. J. Harmsworth Trust, and a Colston Research Scholarship enabled us to secure the services of Dr. Bruce Perry. The Centre was formally opened by Sir Thomas Horder, Bart., in March, 1927, and has occupied itself in various researches now about to be briefly described. Names are omitted, but the writer wishes here to express his deep indebtedness to the Medical and Nursing Staffs as well as to the administration of the Hospital for their continued interest and help.

*Field Research into the Conditions favouring the occurrence of Rheumatic Heart Disease in Childhood.*— The steps taken to organize this research have been detailed from time to time in reports to the *British Medical Journal.* The most recent of these, a report briefly summarizing results as far as this has been possible, has recently been issued by Dr. W. G. Savage,[1]

and there is, therefore, no need to recount the means by which this inquiry was set in motion. All that need be said here is that it has enjoyed the services of over 200 medical practitioners—general practitioners, medical officers of health, school medical officers, and physicians — and that it has been to those concerned in it a signal proof of the capacity of the medical profession to work together in a voluntary organization.

As the inquiry is not yet complete, we must claim only one fact as established. That, however, is so striking as to take a prominent place among the few things that we know for certain about the causation of rheumatic heart disease. It has shown us that during the three years under review (1927–30) Bristol's population of about 400,000 included 750 children with this disease, while the surrounding country with about 1,100,000 inhabitants had 350 of these children. In other words, the incidence in the city was six times as high as in the country. This fact has been carefully examined and tested for errors, but nothing can be found to upset the broad conclusion just stated. Steps are already being taken to see whether Bristol is singular in this respect, or whether the other great cities of Britain are similarly afflicted ; while it is obvious that further inquiries must be made to discover, if possible, what are the reasons for this striking disparity of incidence.

*Histological Studies.*—(*a*) *Rheumatic Heart Disease.* —As a good deal of the work done on this subject was already completed, and indeed published before this Centre came into being, it would be scarcely fair to claim the whole of it as an outcome of the Centre's activities. It is, however, true that its work has confirmed and extended the conclusions already drawn. In particular, the view that had already been put forward, namely that the infective agent enters the cardiac tissues by way of the coronary blood-supply, has been supported by observations of the state of the main trunks of the coronary arteries themselves [2] and of the root of the aorta.[3] Both these areas are damaged by the infection in a majority of cases.

Here we may, perhaps, mention other inquiries made, or in progress, as to lesions of other organs in acute rheumatic infection. One paper on abdominal manifestations [4] supports the view that the peritoneum is occasionally attacked, and work on pulmonary lesions in acute rheumatism and on the distribution of the cerebral changes in chorea is in progress.

(*b*) *Ulcerative Endocarditis.*—Though we have not yet published anything immediately bearing on these infections of the heart, the view already stated, to the effect that we have in this disease an infection of the inner surface of the heart and not of the heart wall itself—an "accidental" implantation of bacteria from the intracardiac blood-stream—has been substantiated. The conclusion to which we are irresistibly driven is that ulcerative endocarditis is for the most part a "terminal" infection, *i.e.* a lesion the very occurrence of which is unfortunately sufficient proof in itself of a fatal failure of resistance, either local or general or both. It cannot be cured, and only very seldom arrested. The only hope lies in prevention of those infections and other agencies which by damaging the endocardium, or letting in an unusual dose of some other virus, lead up to the fatal implantation.

*Cardio-Aortic Syphilis.*—Examination of clinical and histological material led to conclusions [5] which have a bearing not only on the prognosis and treatment of the cardiac lesions but also on the nature of the syphilitic process. It became clear that the view which regarded the heart as one among many organs which, having been saturated by a blood-borne spirochætal infection, suffered damage from an invincible remnant of spirochætes, must be discarded. Apart from gummata—rarely seen in the heart—the only lesion is an aortitis, which injures the heart, not only directly by depriving it of the help that it is entitled to expect from the aortic elasticity, but also by infiltrating the bases of the aortic cusps, which are thereby rendered incompetent, and by narrowing the mouths of the coronary arteries and thus starving the myocardium.

The aortic lesions are lymphogenous, and not hæmatogenous. The virus—whether spirochæta or toxin or both—is brought to the aortic wall by the lymphatics which surround it. From this and from clinical considerations it seems clear that the last phase of the infection, whether it concerns the aorta or the cerebro-spinal axis, or both, is a lymphatic phase. More than that it would be improper to say here, as this is not the place for conjectures.

*Other Infections.* — Specimens of staphylcoccal infection of the heart, of the pericarditis terminating renal disease, and of other infective lesions, have been examined. The first appears to be primarily a focal necrosis of the myocardium which may extend to either of the serous layers. The second, contrary to what one might have expected, seems to be plastic rather than purulent.

*Course and Treatment of Cardiac Infections.*—As a beginning has been made with the treatment of juvenile cardiac rheumatism by what may be called "sanatorium" methods at Winford, it is probable that we shall learn a good deal about ways of distinguishing between activity and quiescence. In the meantime we have tried to discover criteria of activity by means of (*a*) leucocyte counts[6] (*b*) electrocardiography. (This latter investigation entailed a "control" one on children of the same age with normal hearts, the results of which are being published.) It has, however, proved impossible to discover any reliable index of active carditis by these methods. Repeated skiagraphic examinations were also made. These studies are being continued at Winford, and are, therefore, not yet concluded.

A summary has been made of the various means employed in the treatment of ulcerative endocarditis, and although this has not disclosed any new facts, it will be published by way of illustrating the various methods of attack that have been attempted.

*The Ætiology of Cardio-Vascular Disease.*—From the time of its opening the Centre has classified its clinical case records, so far as cardiac disease is concerned, under ætiological headings. The purpose of this was to discover the relative importance of the various morbid influences causing cardiovascular disease. Recently the data thus accumulated were summarized, together with those derived from private practice, and the results formed the basis of a discussion at which similar analyses of experience in other great cities were compared. No gross discrepancies were brought to light. The Bristol figures are set out in the following table:—

*Incidence of Chief Kinds of Organic Disease of Heart expressed in Percentages.*

| *Type.* | *Clinical.* | | | *P.M.* |
|---|---|---|---|---|
| | *Private.* | *Hospital.* | *Total.* | |
| Congenital .. | 1·5 | 2·1 | 1·7 | 0·6 |
| Rheumatic .. | 21·1 | 55·8 | 31·7 | 24·6 |
| Syphilitic .. .. | 3·3 | 8·0 | 4·6 | 12·7 |
| Endocarditis .. | 4·9 | 8·1 | 5·9 | 13·0 |
| Thyrotoxic .. | 6·3 | 8·1 | 6·9 | 0·3 |
| Hyperpietic with Decrescent .. | 57·3 | 13·7 | 44·0 | 46·8 |
| (Systolic B.P. over 200) .. | 14·8 | 2·9 | 11·2 | Cardio-renal 6·7 |

The small proportion of cases ascribed to *congenital defect* is, perhaps, not quite a faithful reflex of the truth. Experiences [7] in a clinic attended by children from the public elementary schools of Bristol have shown that in a school population of about 55,000 there are 116 children who exhibit signs of congenital cardiac anomaly. As only 8 per cent. of these displayed any disability, it is possible that many cases are also overlooked among children not examined medically as a matter of routine. Congenital heart disease has an importance beyond that of the mechanical consequences of the defects, since these are so often the basis of an endocardial infection.

A short paper[8] has been published in which attention has been drawn to the cardiac failure which may terminate *spinal deformity*, the link between the two being furnished by the effects of the deformity on the great vessels which compose the pedicle of the heart.

Another inherent factor of disease that has been examined[9, 10] is that by which the *coronary arteries* of certain families and individuals undergo premature degeneration. The importance of such inquiries is obvious when the apparent increase in the incidence of cardiac infarction is considered. The ætiology of this syndrome has been used[11] to throw light on the meaning of anginal pain and to support the view that this is an ischæmia of the cardiac muscle, usually coronary in its origin.

The importance of *syphilis* as a cause of cardiovascular disease has been noted above. Comparisons indicate that it is rather less important in Britain than on the Continent of Europe and in the United States of America; partly, no doubt, because it is in this country diluted by an excessive number of cases of rheumatic heart disease.

Another of the routine practices of the Centre which has harvested a wealth of knowledge that may be of use some day is that of cutting, in every heart examined, sections of a number of blocks from different parts of the heart. These include the principal portions of the sinu-auricular node and of the auriculo-ventricular bundle.[12] It is a practice that may conceivably furnish a basis for studies in cardiac localization similar to those which have been so fruitful in the brain and spinal cord. We have plenty of material for this kind of work, waiting for someone to use it.

This last remark brings us to a consideration of the future of the Centre. Opened in 1927, on the understanding that the premises used would be available for three years, it is now "carrying on," but steps are being taken, in concert with the Professor of Medicine in the University, to implement the understanding that has existed since its foundation, that it should always be regarded as a part of the Department of Medicine. The chief difficulty by which we are now faced is the inadequacy of that Department. It has no premises in any of our hospitals, and in fact it would be difficult to place it in any. Now, however, that union between our chief hospitals is being accomplished through the medium of the Hospitals' Council, the foundation of a University Hospital, or system of hospitals, with adequate academic departments, is in sight. The Department of Medicine should, perhaps, come first, not only by virtue of seniority, but also and more especially because it is practicable. The building of a block to accommodate medical patients from both Infirmary and Hospital, that will be begun at Winford as soon as funds have been collected, ought to include provision for a department of medicine. The opening of this would furnish a fitting celebration, not only of the centenary of the Bristol Medical School, but also of that of the Bristol General Hospital, and of the bicentenary of the Bristol Royal Infirmary, since it would both commemorate the historical associations of these three, and inaugurate a new era in the life of the Bristol School of Medicine. It might, for example, be possible to arrive at an understanding by which the past and present students of that School agreed to provide the necessary equipment, if the personnel were found by the University and the premises by the Hospitals' Council. Some such provision must be made for research in medicine, and it is in the achievement of this object that the University Centre of Cardiac Research hopes to find itself absorbed.

REFERENCES.

[1] Savage, W. G., *Brit. Med. Jour.*, 1931, ii., July 18th (Supplement).

[2] Perry, C. B., *Quart. Jour. Med.*, 1930, xxiii. 241.

[3] Giraldi, J. J. J., *Bristol Med.-Chir. Jour.*, 1929, xlvi. 145.

[4] Giraldi, J. J. J., *Arch. Dis. in Childhood*, 1930, v. 379.

[5] Coombs, C. F., *Lancet*, 1930, ii. 227, 281 and 333 ; *Brit. Med. Jour.*, 1930, ii. 893.

[6] Perry, C. B., *Arch. Dis. in Childhood*, 1929, iv. 247.

[7] Perry, C. B., *Bristol Med.-Chir. Jour.*, 1931, xlviii. 41.

[8] Coombs, C. F., *Brit. Jour. Surg.*, 1930, xviii. 326.

[9] Perry, C. B., and Herapath, C.E. K., *Brit. Med. Jour.*, 1930, i. 685.

[10] Perry, C. B., and Hughes, F. W. T., *Bristol Med.-Chir. Jour.*, 1929, xlvi. 219.

[11] Coombs, C. F., *Quart. Jour. Med.*, 1930, xxiii. 233.

[12] Coombs, C. F., *Proc. Brit. Assoc. for Advancement of Science*, 1930 (Physiol.).

